# A Unifying Model for Capture–Recapture and Distance Sampling Surveys of Wildlife Populations

**DOI:** 10.1080/01621459.2014.893884

**Published:** 2015-04-22

**Authors:** D. L. Borchers, B. C. Stevenson, D. Kidney, L. Thomas, T. A. Marques

**Keywords:** Abundance estimation, Acoustic survey, Closed population, Measurement error, Visual survey

## Abstract

A fundamental problem in wildlife ecology and management is estimation of population size or density. The two dominant methods in this area are capture–recapture (CR) and distance sampling (DS), each with its own largely separate literature. We develop a class of models that synthesizes them. It accommodates a spectrum of models ranging from nonspatial CR models (with no information on animal locations) through to DS and mark-recapture distance sampling (MRDS) models, in which animal locations are observed without error. Between these lie spatially explicit capture–recapture (SECR) models that include only capture locations, and a variety of models with less location data than are typical of DS surveys but more than are normally used on SECR surveys. In addition to unifying CR and DS models, the class provides a means of improving inference from SECR models by adding supplementary location data, and a means of incorporating measurement error into DS and MRDS models. We illustrate their utility by comparing inference on acoustic surveys of gibbons and frogs using only capture locations, using estimated angles (gibbons) and combinations of received signal strength and time-of-arrival data (frogs), and on a visual MRDS survey of whales, comparing estimates with exact and estimated distances. Supplementary materials for this article are available online.

## 1 INTRODUCTION

Estimating animal population density is crucial for successful and efficient management and conservation of wildlife resources. As a complete census is rarely feasible, this usually requires survey sampling, most often using one of the two dominant survey methods: capture–recpature (CR) or distance sampling (DS; see Schwarz and Seber 1999; Borchers, Buckland, and Zucchini 2002; Williams, Nichols, and Conroy 2002; Royle and Dorazio 2008, for overviews of methods). In CR, a series of detectors (e.g., traps or cameras) are deployed on multiple sampling occasions. The resulting “capture history” of occasions on which each uniquely identified animal was detected is used to estimate the probability of detection, and hence account for undetected animals. DS requires only a single survey occasion and uses the distances of detected animals from detectors to estimate the detection probability and hence account for animals missed.

Both methods sample a subset of the area occupied by the population of interest and both require some measure of effective area sampled to estimate animal density. When detection is not certain, effective area sampled is obtained by integrating under an estimated detection probability surface. DS methods estimate the detection probability surface by using observed distances to detections to estimate detection probability as a function of distance from detector. CR methods have until recently had no statistically rigorous method for estimating density, but this changed with the advent of spatially explicit capture–recapture (SECR) methods (Efford 2004; Borchers and Efford 2008; Royle and Young 2008; Royle et al. 2013a). SECR data do not include distances to animal locations; instead SECR methods use the distances between detectors at which animals are (and are not) detected to estimate a distance-based detection probability surface.

As it involves a distance-based detection function, SECR is closer to DS than is traditional CR, and in fact SECR methods have borrowed detection function forms from DS. At the same time, there have been developments in DS that bring it closer to CR methods. For example, standard DS methods have been extended to use two independent observers, generating capture history as well as DS data—a method known as mark recapture distance sampling (MRDS; Manly, McDonald, and Garner 1996; Borchers, Zucchini, and Fewster 1998).

In this article, we unify DS and CR methods and in doing so create a class of model that includes a range of models that can be viewed as hybrids of them. Examples include MRDS surveys with distance measurement error and SECR surveys that contain additional information about animal location, such as received acoustic signal strength (SS), precise time of acoustic detection, or estimated bearing to detected animals. We demonstrate the new class through a series of applications to both real and simulated datasets.

## 2. MOTIVATING PROBLEMS

### 2.1 Gibbon Survey

Gibbons are difficult to detect visually in forest but can be detected quite easily acoustically when they make territorial calls. An acoustic survey with human detectors, of northern yellow-cheeked gibbon (*Nomascus annamensis*) was conducted in northeastern Cambodia by Conservation International in 2010. The design involved three people standing in a line spaced approximately 500 m apart, recording estimated angles to all gibbon groups they heard. Observers who detected a group comprise the group’s capture history, while the estimated angles to detected groups provide additional data on group location. Use of the additional data is shown to improve inference.

### 2.2 Frog Survey

An acoustic survey of Lightfoot’s moss frog (*Arthroleptella lightfooti*) in a water seepage on Table Mountain, South Africa, was conducted using six microphones in a roughly rectangular arrangement. The survey is similar to the gibbon survey in that spatial capture histories consist of the locations of detectors (microphones) at which each vocalization (frog click) was detected. The time difference of arrival (TDOA) of the same click at different detectors and the received SS at each detector provide additional data on animal location. Each of the additional data types improves inference in this case.

### 2.3 Minke Whale Survey

As part of the 2001 North Atlantic Sightings Survey (NASS 2001; see Pike et al. 2009, for details), two independent observers surveyed the same region of sea simultaneously from an aircraft, recording estimated distances to detected whale cues (blows). The detectors (the observers) were at the same location, and capture histories indicate which observer(s) detected each cue. Having the detectors at the same location has implications for SECR analysis that we expand upon below. Additional data on whale location are contained in the estimated distances to detected cues, even though they are subject to measurement error. Use of these data is shown to substantially reduce density estimation bias.

## 3. THE MODEL

### 3.1 Animal Location

We use a generic notion of animal location, specified via Cartesian coordinates $x=(x_{1},x_{2})$. In DS surveys, x is the actual location of an animal at the time of the survey. If an animal moves during the survey its location x represents the average of its positions over the survey. In the context of trapping studies, these locations have variously been called, “home range centers,” “centroids,” and “activity centers” (Borchers and Efford 2008; Royle and Young 2008; Royle et al. 2009a). Ideally, we would like to observe x, but this may not be possible. Below we derive a likelihood function that accommodates situations in which location is observed, in which it is partially observed or observed with error, and in which only locations of the detectors are observed. We develop the likelihood for SECR surveys without any information on animal locations other than the spatial capture history, and then extend this to include location observation data.

### 3.2 Probability Model and Likelihood

Consider a survey of a region with surface area *A* in which *K* detectors are deployed on *S* occasions. Following Borchers and Efford (2008), we assume that animals are independently distributed in this region according to a nonhomogeneous Poisson process (NHPP) with parameter vector φ and intensity D(x;φ) at x. We denote the probability that an animal at x is detected by at least one detector on the survey as $p_{\cdot }\left(x;\theta \right)$, with unknown parameter vector θ. It follows that the locations of detected animals, $X=(x_{1},...,x_{n})$, are realizations of a filtered NHPP with intensity $D\left(x;\varphi \right)p_{\cdot }\left(x;\theta \right)$ at x.

We construct a probability model for the outcomes of a survey via a product of conditional probabilities, which are developed below. The first component of the model is the probability of detecting *n* animals: P(n;φ,θ). The second is the probability density function (pdf) of animal locations, X, conditional on detection, which we write as $f_{X}\left(X;\varphi ,\theta \right)$.

The third component is the probability of observing the capture histories Ω, conditional on detections and detected animal locations X, which we write as P(Ω∣X;θ). Here $\Omega =(\omega_{1},...,\omega_{n})$, where $\omega_{i}$ is the capture history of the *i*th animal. The joint pdf of *n*, X, and Ω is then

(1) $$\begin{array}{ccc} f_{n,X,\Omega }\left(X,n,\Omega ;\varphi ,\theta \right) & = & P\left(n;\varphi ,\theta \right)f_{X}\left(X;\varphi ,\theta \right)P\left(\Omega |X;\theta \right). \end{array}$$

We now expand upon each of the terms on the RHS of this equation, after which we add a term for (possibly noisy) observations of animal locations.

Note that our model assumes that each animal has a single x for the survey. This does not mean that animals do not move, just that x is the center of activity over the whole survey if they do move. We discuss this further in Section 5.

#### Capture History Given Location. P(Ω∣X;θ)

3.2.1 

We define an indicator variable ω_*iks*_ that is 1 if animal *i* is detected by detector *k* on occasion *s* and is 0 otherwise, so that the capture history of animal *i* on occasion *s* is $\omega_{is}=\left(\omega_{i1s},...,\omega_{iKs}\right)$ and its full capture history is $\omega_{i}=\left(\omega_{i1},...,\omega_{iS}\right)$. It is convenient to define two indicator variables derived from ω_*iks*_, as follows: let ω_*i* · *s*_ = 1 if animal *i* was detected on occasion *s* and ω_*i* · *s*_ = 0 otherwise, and ω_*i* · ·_ = 1 if animal *i* is detected at all and ω_*i* · ·_ = 0 otherwise. Letting *B*(ω, *p*) indicate a Bernoulli probability mass function for ω, with parameter *p*, we can write P(Ω∣X;θ) as

(2) $$\begin{array}{ccc} & & \;P(\Omega |X;\theta )\\ & & \;\;=\prod_{i=1}^{n}\frac{\prod_{s=1}^{S}B\left(\omega_{i\cdot s},p_{\cdot s}\left(x_{i};\theta \right)\right)\text{Pr}{\left(\omega_{is}|\omega_{i\cdot s}=1;\theta \right)}^{\omega_{i\cdot s}}}{p_{\cdot }\left(x_{i};\theta \right)} \end{array}$$

where $p_{\cdot s}\left(x_{i};\theta \right)=1-\prod_{k=1}^{K}\left\{1-p_{ks}\left(x_{i};\theta \right)\right\}$ is the probability that animal *i* at $x_{i}$ is detected on occasion *s*, $p_{ks}\left(x_{i};\theta \right)$ is the probability that animal *i* is detected by detector *k* on occasion *s*, and $p_{\cdot }\left(x_{i};\theta \right)=1-\prod_{s=1}^{S}\left\{1-p_{\cdot s}\left(x_{i};\theta \right)\right\}$ is the inclusion probability for animal *i*, that is, the probability that it is detected at all. $P(\omega_{is}|\omega_{i\cdot s}=1;\theta )$ is the probability that on occasion *s* detected animal *i* has capture history $\omega_{is}$. This probability is different for proximity detectors (which detect animals without detaining them) and detectors that hold animals until the end of the occasion. Appendix A contains the details for each of these cases. It is also shown in this Appendix that in the case of proximity detectors with a single occasion and any survey with a single detector and multiple occasions, P(Ω∣X;θ) is identical to the conditional likelihood of Huggins (1989).

So if the $x_{i}$'s were observed, we could estimate abundance using the conditional likelihood approach of Huggins (1989), with x as the observed covariate vector. This implies that (unlike conventional CR) estimation is possible from multiple detectors on one occasion with proximity detectors, as recaptures within occasion are possible. (Efford, Borchers, and Byrom 2009a, first noted this fact.)

Because animal location (x) is not observed on conventional CR studies (only locations of capture are observed), we cannot take the approach of Huggins (1989). But the location covariate x is observed on MRDS surveys, which involve a single occasion (*S* = 1) and typically two observers (*K* = 2), acting as independent detectors, recording locations of detections. In this case, we could use the approach of Huggins (1989). This is, however, seldom done because on MRDS and other DS surveys with randomized sampler locations, animal locations in the vicinity of detectors can be treated as random variables with a known pdf determined by design (namely a uniform distribution in the plane) and Borchers (1996) showed that using this pdf of locations in estimation usually improves MRDS estimator properties. Hence, the estimator of Huggins (1989), which conditions on locations, is not optimal for MRDS estimation and is generally not used for MRDS data. Instead MRDS inference is based on likelihood functions that treat X as random. These involve the conditional distribution of animal locations given detection, $f_{X}\left(X;\varphi ,\theta \right)$, which we now consider in more detail.

#### Animal Locations, Given Detection. $f_{X}\left(X;\varphi ,\theta \right)$

3.2.2 

As noted above, MRDS methods assume an independent uniform distribution of animals within detectable range (Borchers, Zucchini, and Fewster 1998). This distribution is consistent with animals being distributed according to a homogeneous Poisson process (HPP) in the plane. We make the more general assumption that animals occur according to an NHPP, with intensity D(x;φ) at x. As an animal at x is detected with probability $p_{\cdot }\left(x_{i};\theta \right)$, it follows that detected animals occur according to a filtered NHPP with intensity $D\left(x_{i};\varphi \right)p_{\cdot }\left(x_{i};\theta \right)$. The pdf of x given detection is obtained using Bayes’ theorem as $f_{x}\left(x_{i};\varphi ,\theta \right)=D\left(x_{i};\varphi \right)p_{\cdot }\left(x_{i};\theta \right)/\lambda \left(\varphi ,\theta \right)$, where $\lambda \left(\varphi ,\theta \right)=\int_{R^{2}}D\left(x;\varphi \right)p_{\cdot }\left(x;\theta \right)\;dx$. Assuming independent detections, we have $f_{X}\left(X;\varphi ,\theta \right)=\prod_{i=1}^{n}f_{x}\left(x_{i};\varphi ,\theta \right)$. The same $f_{X}\left(X;\varphi ,\theta \right)$ is obtained if we treat the number of animals in the area as fixed at *N* and assume that these animals are located independently in space with probability density $\pi \left(x;\varphi \right)=D\left(x;\varphi \right)/\int_{R^{2}}D\left(x;\varphi \right)\;dx$ at x.

#### Number of Detections. P(n;φ,θ)

3.2.3 

If animals are independently distributed in the plane according to an NHPP with parameter vector φ and intensity D(x;φ) at x, and they are independently detected with probability $p_{\cdot }\left(x;\theta \right)$, it follows that *n*, the number of detected animals, is a Poisson random variable with rate parameter λ(φ,θ). If the number of animals in the area is a fixed number *N*, then *n* is a binomial random variable with parameters *N* and $p_{\cdot }=\int_{R^{2}}\pi \left(x;\varphi \right)p_{\cdot }\left(x;\theta \right)\;dx$.

#### Location Observation Given Capture History. f(Y∣X,Ω;γ)

3.2.4 

Suppose now that in addition to observing ω_*iks*_ for animal *i* on occasion *s*, we also observe a vector $y_{iks}=\left(y_{iks1},...,y_{iksM}\right)$ containing *M* different kinds of data, each of which is a noisy observation of animal location. An example with *M* = 2 is an acoustic survey in which detectors are microphones and SS (*y*_*iks*1_) and time of arrival (*y*_*iks*2_) of the sound at a microphone are recorded. Writing the set of all observations $y_{iks}$ as Y, we write the conditional pdf of Y given X as $f_{Y|X\Omega }\left(Y|X,\Omega ;\gamma \right)$, where γ is a vector of parameters to be estimated. In the models we consider, the $y_{iks}$'s are conditionally independent, given X. In general, *y_iksm_* may affect detection probability, and in this case $p_{ks}\left(x;\theta \right)$ must be replaced by $p_{ks}\left(x,y_{iks};\theta ,\gamma \right)$ in all of the above, and P(n;φ,θ), $f_{X}\left(X;\varphi ,\theta \right)$, P(Ω∣X;θ) become P(n;φ,θ,γ), $f_{X}\left(X;\varphi ,\theta ,\gamma \right)$, P(Ω∣X;φ,θ,γ). (See Efford, Dawson, and Borchers 2009b, and below.)

Following Efford, Dawson, and Borchers (2009b), we model the expected value of the random variable *y_m_* (dropping the *iks* subscript for brevity here), given x, as $E\left(y_{m}|x\right)=\mu_{m}\left(x\right)=g_{m}^{-1}\left(\beta_{0m}+\beta_{1m}h_{mk}\left(x\right)\right)$. Here *g_m_* is a link function, $\beta_{m}=\left(\beta_{0m},\beta_{1m}\right)$ is a component of γ and the “proxy function” $h_{mk}\left(x\right)$ returns the component of location for which *y_m_* is a proxy, at detector *k*. For example, if *y_m_* is either the observed distance from detector to animal or the received SS, then $h_{mk}\left(X\right)$ is the true distance from detector *k* to the animal.

### 3.3 The Likelihood Function

The joint density of *n*, X, Ω and Y is

(3) $$\begin{array}{ccc} \;f(n,X,\Omega ,Y;\varphi ,\theta ,\gamma ) & = & P(n;\varphi ,\theta ,\gamma )\\ & & \;\times \;f_{X\Omega Y}\left(X,\Omega ,Y|n;\varphi ,\theta ,\gamma \right), \end{array}$$

where $f_{X\Omega Y}\left(X,\Omega ,Y|n;\varphi ,\theta ,\gamma \right)$ is the product of $f_{X}\left(X;\varphi ,\theta ,\gamma \right)$, P(Ω∣X;φ,θ,γ) and $f_{Y|X\Omega }\left(Y|X,\Omega ;\gamma \right)$. In general, X is not observed and this density cannot therefore be evaluated. We obtain our likelihood by marginalizing over X in Equation (3):

(4) $$\begin{array}{ccc} \;L(\varphi ,\theta ,\gamma |n,\Omega ,Y) & = & P(n;\varphi ,\theta ,\gamma )\\ & & \;\times \;\int_{R^{2}}f_{X\Omega Y}\left(X,\Omega ,Y;\varphi ,\theta ,\gamma \right)\;dX \end{array}$$

and we estimate φ,θ,γ by maximizing this equation with respect to φ,θ,γ. We obtain interval estimates using the inverse of the negative Hessian matrix, which is obtained from numerical maximization of the likelihood. Model selection can be done using AIC or any other likelihood-based method.

#### 3.3.1 Estimating Animal Location

Given estimates $\hat{\varphi }$, $\hat{\theta },$ and $\hat{\gamma }$, animal locations can be estimated from Ω,Y by application of Bayes’ Theorem as follows (omitting $\hat{\varphi }$, $\hat{\theta }$, and $\hat{\gamma }$ for brevity and indicating estimates by “hats” on functions):

(5) $$\begin{array}{ccc} \;{\hat{f}}_{X|\Omega Y}\left(X|\Omega ,Y\right) & = & \frac{{\hat{f}}_{Y|X\Omega }\left(Y|X,\Omega \right)\hat{P}\left(\Omega |X\right){\hat{f}}_{X}\left(X\right)}{\int_{R^{2}}{\hat{f}}_{Y|X\Omega }\left(Y|X,\Omega \right)\hat{P}\left(\Omega |X\right){\hat{f}}_{X}\left(X\right)\;dX}. \end{array}$$

Besides being of possible inherent interest, the pdf of animal locations, ${\hat{f}}_{X|\Omega Y}\left(X|\Omega ,Y\right)$, is useful for illustrating the effect of the location observation data Y on the precision of location estimation, and we use it primarily for this purpose below.

## 4. ANALYSES OF MOTIVATING PROBLEMS

**Figure 1:**
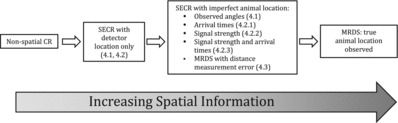
A continuum of increasingly spatially resolved capture–recapture models. Numbers in brackets correspond to subsections of the article.

The continuum of increasingly spatially resolved spatial sampling models covered in this article is illustrated in Figure 1. SECR models without location observations Y are obtained by omitting $f_{Y|X\Omega }\left(Y|X,\Omega ;\gamma \right)$ from the model. Detection probability of an animal at distance zero from detectors (denoted $p_{ks}\left(x^{\left(k\right)};\theta \right)$, with $x^{\left(k\right)}$ being the location of the *k*th detector) may be constrained to be 1 or not, depending on the application. DS and MRDS models are obtained by defining $f_{Y|X\Omega }\left(Y|X,\Omega ;\gamma \right)$ to be unity at Y=X and zero elsewhere. MRDS models generally have *K* = 2 and *S* = 1 and allow $p_{ks}\left(x^{\left(k\right)};\theta \right)<1,$ while conventional DS models have *K* = 1, *S* = 1 and define $p_{ks}\left(x^{\left(k\right)};\theta \right)=1$.

**Figure 2:**
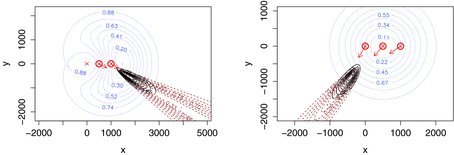
Example location estimates, given capture, of two different gibbons. Detectors are crosses; circled detectors are those that detected the gibbon call. Arrows show estimated angles to detections. Dotted lines are the contours of the estimated probability of the group being contained within the contour, given only the spatial capture history data Ω. Dashed lines are estimated contours, given only observed angles to detections. Solid lines are estimated contours, given capture history and angles.

All the case studies below involve proximity detectors and a single occasion (so we omit subscript *s*), but the methods apply equally to multi-catch traps and multiple occasions. We do not include any covariates or individual random effects (other than x) in our applications for brevity and because our emphasis is on illustration of the effects of adding supplementary data. See Discussion for more on covariates.

All analyses and plots were done with the R library admbsecr, written by authors of this article (see online supplementary material).

### 4.1 Gibbon Survey: SECR With Estimated Angles

#### 4.1.1 The Model

Recall that the detectors are observers standing in a line spaced approximately 500 m apart (see Figure 2), recording estimated angles to gibbon groups they heard. We use SECR methods to estimate the density of calling groups from the locations of the observers who detected the group, both with and without the angle data.

Here *S* = 1 and we model the probability of detecting animal *i* with location $x_{i}$ in trap *k* on this occasion as $p_{k1}\left(x_{i};\theta \right)=\exp\left\{-d_{k}{\left(x_{i}\right)}^{2}/\left(2\theta^{2}\right)\right\}$, where θ≡θ, $d_{k}\left(x_{i}\right)$ is the distance from observer *k* (located at coordinates $z_{k}=\left(z_{k1},z_{k2}\right)$) to animal *i* at $x_{i}=\left(x_{i1},x_{i2}\right)$: $d_{k}\left(x_{i}\right)=\sqrt{{\left(z_{k2}-x_{i2}\right)}^{2}+{\left(z_{k1}-x_{i1}\right)}^{2}}$. We assume an HPP for animal locations with D(x;φ)=φ.

Supplementary data comprise recorded angles to animals, so *M* = 1 and, dropping subscripts *s* and *m* for brevity, we let *y_ik_* denote the recorded angle between animal *i* and detector *k*, with respect to some reference direction. The proxy function $h_{k}\left(x_{i}\right)$ is the inverse tangent of (*z*_*k*2_ − *x*_*i*2_)/(*z*_*k*1_ − *x*_*i*1_)). We assume an identity link in the expectation function so that $E\left(y_{m}|x\right)=\beta_{0}+\beta_{1}h_{k}\left(x\right)$, and we assume angles are estimated without bias at all distances so that β_0_ = 0 and β_1_ = 1. A von Mises distribution with concentration parameter γ is used to model the angle observation error (γ≡γ). With independent angle observation errors,

(6) $$\begin{array}{ccc} \;f(Y|X,\Omega ;\gamma ) & = & \prod_{i=1}^{n}\prod_{k=1}^{K}{\left[\frac{\exp\left\{\gamma \cos\left[y_{ik}-h_{k}\left(x_{i}\right)\right]\right\}}{2\pi I_{0}\left(\gamma \right)}\right]}^{\omega_{ik}}, \end{array}$$

where *I*_0_( ) is the modified Bessel function of order 0.

#### 4.1.2 Results

A total of 123 detections of 77 calls were made. Using only capture histories (Ω), the density of calling gibbon groups is estimated to be 0.83 groups per square kilometer, with a coefficient of variation (CV) of 44%, while using Ω and Y it is estimated to be 0.32 with CV of 23%. The differences arise as a consequence of the estimated detection function scale parameter θ being much smaller when only Ω is used ($\hat{\theta }$ = 754 m; CV = 23%) than when Y is also used ($\hat{\theta }$ = 1248 m; CV = 11%).

**Figure 3:**
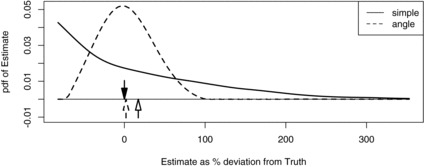
Smoothed simulated sampling distributions of estimated gibbon call density when only spatial capture history is used in estimation (“simple”) and when capture history and observed angles are used (“angle”). The down arrow marks true (simulated) density, the horizontal axis is percentage deviation from true density, and the up arrows are the means of the sampling distributions.

To investigate the cause of the differences we plotted estimated locations of calling groups using Equation (5), and we conducted a simulation study (with 500 simulations) in which true parameter values were equal to those estimated using Ω and Y. Illustrative examples of location contours are shown in Figure 2 and the simulated sampling distributions of the two estimators is shown in Figure 3.

The utility of angle data is apparent in Figure 2 in the form of much tighter contours when Ω and Y are used than for Ω alone. It is also apparent in Figure 3, which shows the “simple” estimator using only Ω to be biased (by about 15%), very much more dispersed and with a mode far below truth (“truth” being the density used in simulating). (Note that with three detectors there are only seven possible capture histories and hence the simple SECR model will estimate all animals to be at one of only seven locations, while with the angle data, an infinite number of locations is possible.)

Part of the problem is poor design: with detectors spaced only 500 m apart and scale parameter θ = 1248 the simple estimator has no information on how detection probability varies at distances greater than 1000 m—because detections are never more than 1000 m apart. The angle data overcome this limitation: use of Y improves estimation.

### 4.2 Frog Survey: SECR With Arrival Times and Signal Strength

**Figure 4:**
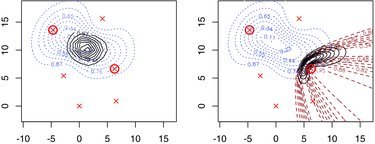
Estimated location contours given capture history and SS (left) and capture history and TDOA (right), of a click. Detectors are crosses; circled detectors are those that detected the frog click. Dotted lines are the contours of the probability density of frog location given only spatial capture history data Ω. Dashed lines in the right plot are contours given only TDOA. Solid lines are contours of location given capture history and SS (left) or capture history and TDOA (right).

In this case, we have multivariate location data Y, comprising the TDOA and SS of detected frog clicks. We have one occasion (*S* = 1) and the arrangement of the six microphones (*K* = 6) is shown in Figure 4.

We compare estimators using SECR methods with no location observations, using TDOA data, using SS data, and using both. We use the same forms for $p_{k}\left(x_{i};\theta \right)$, $d_{k}\left(x_{i}\right)$, and D(x;φ) as were used in the gibbon survey. Models for TDOA data and SS are specified below, followed by analysis and simulation results for each case.

#### 4.2.1 TDOA Observation

As we have only one kind of supplementary location data (*M* = 1), we omit the *m* subscript and we let *y_ik_* denote the time of arrival of the *i*th clicks at detector *k*. The proxy function $h_{k}\left(x_{i}\right)$ is the distance function $d_{k}\left(x_{i}\right)$ (in meters) used above. We assume normal errors in time of arrival, and constant variance σ^2^_*t*_ of this error at all microphones, which is consistent with randomness in time of arrival being dominated by measurement error. We use an identity link so that $E\left(y_{ik}|x_{i}\right)=\beta_{0i}+\beta_{1}h_{k}\left(x_{i}\right)$, where β_0*i*_ is the time the *i*th sound was generated and β_1_ is the inverse of the speed of sound in air (in meters per second).

The time clicks are made is uninformative about location, as a click made at distance d(x) at time β_0_ has the same expected arrival time as one made at distance $d\left(x\right)+c/\beta_{1}$ at time β_0_ − *c*, for any *c*. The β_0*i*_'s are what (Millar 2011, pp. 188–189) called incidental parameters, and to eliminate them we can base inference on the likelihood of time *differences* of arrival (TDOAs) from the mean arrival time, conditional on the mean arrival time:

(7) $$\begin{array}{ccc} & & f_{Y|X\Omega }\left(Y|X,\Omega ;\gamma \right)\\ & & \;\propto \prod_{i=1}^{n^{+}}{\left(2\pi \sigma_{t}^{2}\right)}^{(1-m_{i})/2}\exp\left\{\sum_{k=1}^{m_{i}}\frac{{\left(\delta_{k}\left(x_{i}\right)-{\bar{\delta }}_{i}\right)}^{2}}{-2\sigma_{t}^{2}}\right\}, \end{array}$$

where *n* ^+^ is the number of clicks detected on more than one microphone, *m_i_* is the number of microphones on which the *i*th of these was detected, $\gamma \equiv \sigma_{t}^{2}$, $\delta_{k}\left(x_{i}\right)=y_{ik}-E\left(y_{ik}|x_{i}\right)$, and ${\bar{\delta }}_{i}=\frac{1}{m_{i}}\sum_{k=1}^{m_{i}}\delta_{k}\left(x_{i}\right)$. The same likelihood can be obtained using a marginal approach, treating the β_0_s as random effects (see online supplementary material). For this reason, and for brevity, we do not explicitly show the conditioning on ${\bar{\delta }}_{i}$s on the LHS of the equation.

#### 4.2.2 Signal Strength (SS) Observation

The ideas of this section are taken from Efford, Dawson, and Borchers (2009b). *M* = 1 and we let *y_ik_* denote the received SS at detector *k*. The proxy function $h_{k}\left(x_{i}\right)$ is as above and we model the expectation as $E\left(y_{ik}|x_{i}\right)=\beta_{0}+\beta_{1}h_{k}\left(x_{i}\right)$, where β_0_ is the mean SS of clicks and β_1_ is a parameter quantifying SS loss with propagation distance. (We also tried a log link, $E\left(y_{ik}|x_{i}\right)=\exp\left\{\beta_{0}+\beta_{1}h_{k}\left(x_{i}\right)\right\}$, but this was found to be inferior in terms of AIC: ΔAIC = 18.) As with the time of arrival model, we assume that *y_ik_* is normally distributed with constant variance, σ^2^_*s*_, but unlike the time of arrival model, we estimate β_0_ and β_1_ in addition to σ^2^_*s*_, so that $\gamma =(\beta_{0},\beta_{1},\sigma_{s}^{2})$. In addition, because signals weaker than some specified strength *c* are filtered out at the acoustic processing stage, detection probability depends on received SS. We can write the probability of microphone *k* detecting signal *i* made at a distance $d(x_{i})$ from it as $p_{k1}\left(x,y_{k};\theta ,\gamma \right)=1-F_{k}\left(c;x_{i},\gamma \right)$, where $F_{k}\left(c;x_{i},\gamma \right)$ is the cumulative distribution function (CDF) of a normal random variable with mean $\exp\{\beta_{0}+\beta_{1}h_{k}\left(x_{i}\right)\}$ and variance σ^2^_*s*_, evaluated at *c*. Then,

(8) $$\begin{array}{ccc} f_{Y|X\Omega }\left(Y|X,\Omega ;\gamma \right) & = & \prod_{i=1}^{n}\prod_{k=1}^{m_{i}}\frac{N_{k}\left(y_{ik};x_{i},\gamma \right)}{1-F_{k}\left(c;x_{i},\gamma \right)}, \end{array}$$

where *m_i_* is as before, the number of microphones on which click *i* was detected and $N_{k}\left(y_{ik};x_{i},\gamma \right)$ is a normal pdf with mean $\exp\{\beta_{0}+\beta_{1}h_{k}\left(x_{i}\right)\}$ and variance σ^2^_*s*_, evaluated at *y_ik_*.

#### 4.2.3 TDOA and Signal Strength (SS) Observation

In this case, *M* = 2 and we let $y_{ik}=\left(y_{ik1},y_{ik2}\right)$ where *y*_*ik*1_ is the time of arrival and *y*_*ik*2_ is the received SS of click *i* at detector *k*. Both $h_{1k}\left(x_{i}\right)$ and $h_{2k}\left(x_{i}\right)$ are the distance function $d_{k}\left(x_{i}\right)$ and we assume the same models as above so that $E\left(y_{ik}|x_{i}\right)=\left(\beta_{01}+\beta_{11}h_{1k}\left(x_{i}\right),\exp\left\{\beta_{02}+\beta_{12}h_{2k}\left(x_{i}\right)\right\}\right)$, $\gamma =(\beta_{11},\sigma_{t}^{2},\beta_{02},\beta_{12},\sigma_{s}^{2})$, and assuming *y*_*ik*1_, *y*_*ik*2_ to be independent we have

(9) $$\begin{array}{ccc} & & f_{Y|X\Omega }\left(Y|X,\Omega ;\gamma \right)\\ & & \;=\prod_{i=1}^{n^{+}}\frac{{\left(2\pi \sigma_{t}^{2}\right)}^{(1-m_{i})/2}}{2T\sqrt{m_{i}}}\exp\left\{\sum_{k=1}^{m_{i}}\frac{{\left(\delta_{k}\left(x_{i}\right)-{\bar{\delta }}_{i}\right)}^{2}}{-2\sigma_{t}^{2}}\right\}\\ & & \;\times \;\prod_{i=1}^{n}\prod_{k=1}^{m_{i}}\frac{N_{k}\left(y_{ik};x_{i},\gamma \right)}{1-F_{k}\left(c;x_{i},\gamma \right)}. \end{array}$$

#### 4.2.4 Comparison of Estimates With and Without TDOA, SS

A total of 590 detections of 345 frog clicks were made. Using SECR only, the click density is estimated to be 152.1 clicks per hectare per minute, with standard error 10.6 (CV = 7.0%). When SS is used these are reduced to 148.9 and 8.9 (CV = 6.0%), when TDOA is used they are reduced to 134.5 and 9.5 (CV=7.1%), and when both SS and TDOA are used, they reduce to 125.7 and 8.0 (CV = 6.4%). While both SS and TDOA reduce the point estimate of density and its standard error, the effect of SS on the point estimate is weaker. Investigation at the individual click level revealed that point estimates of click locations from the TDOA+SS model tended to agree well with those from the simple SECR model (but were more precise), while those from SECR+TDOA often differed substantially. Figure 4 shows an example for a specific click. The average difference in received SS for individual clicks was less than 2% of its mean value and it may be that the distances between microphones were too small for the contrast in received SS to be very informative about location. The same is not true of TDOA.

**Figure 5:**
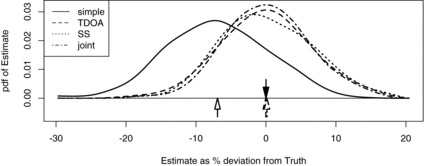
Smoothed simulated sampling distributions of estimated frog click density using only spatial capture history (“simple”), using capture history and time of arrival (“TDOA”), using capture history and signal strength (“SS”), and using capture history, time of arrival and signal strength (“joint”). The down arrow marks true density, the horizontal axis is percentage deviation from true density, and the up arrows are the means of the sampling distributions, expressed as percentage deviation from truth (some are almost coincident).

We investigate estimator properties by simulation (500 simulations), using the parameter estimates from the SECR+SS+TDOA model as truth and mean sample size equal to that observed on the survey. Simulated sampling distributions are shown in Figure 5. As expected, the addition of SS or TDOA reduces bias and improves precision, and there is a further small improvement in precision when both SS and TDOA data are used: the CVs for the SECR, SECR+SS, SECR+TDOA, and SECR+SS+TDOA models are 7.9%, 6.8%, 6.8%, and 6.1%, respectively.

**Figure 6:**
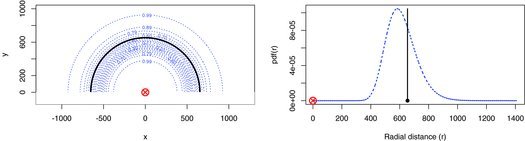
Estimated location contours (dotted) given capture history and recorded location (solid) of a whale detected by one of the two detectors. Contours are such that 100α% of the density falls between the two contours marked α. The left plot shows locations in perpendicular and forward distance space, the right curve shows it in radial distance space. Detectors are crosses.

### 4.3 Whale Survey: MRDS With Estimated Distances

#### 4.3.1 The Model

We estimate the number of minke whale cues per hectare over the sampling period from 71 detections obtained on the aerial cue-counting component of the NASS 2001 survey. *K* = 2 as there were two detectors and *S* = 1 as they made one pass over animals. Standard SECR methods cannot be applied in this case because a distance-dependent detection function cannot be estimated from detectors at a single location. But with the addition of estimated distances to detections (*y_ik_* for observer *k*’s estimate of distance to cue *i*), estimation is possible.

MRDS survey models treat distances as being observed without error (see Borchers et al. 2009; Laake et al. 2011, for cue counting and point transect examples); our model readily allows distance measurement error to be incorporated in MRDS inference, estimating measurement error from the pairs of recorded distances of the two observers to recaptures, simultaneously with density and detection function parameters. In this survey, measurement error is substantial, as can be seen from Figure 6. We estimate cue density allowing probability of detection at distance zero to be less than unity, both with and without the assumption of no measurement error. Were we to enforce certain detection at distance zero, we would have conventional distance sampling (CDS) models with and without measurement error. (See Borchers et al. 2010, for references to CDS models with measurement error.)

Following standard practice for DS surveys, we assume an independent uniform distribution of animals in the plane (Buckland et al. 2001) and hence use a HPP for animal locations with D(x;φ)=φ. This leads to the usual cue-counting pdf for radial distances of detected animals (see online supplementary material). We found it necessary to introduce detector-specific detection function parameters as one detector was far more efficient than the other. We use $p_{k1}\left(x_{i};\theta_{k}\right)={\text{logit}}^{-1}\left(\theta_{k2}\right)\exp\left\{-d_{k}{\left(x_{i}\right)}^{2}/\left(2\theta_{k1}^{2}\right)\right\}$, where $\theta_{k}=\left(\theta_{k1},\theta_{k2}\right)$ (*k* = 1, 2) and $\theta =(\theta_{11},\theta_{12},\theta_{21},\theta_{22})$. The proxy function $h_{1k}\left(x_{i}\right)$ is the distance function $d_{k}\left(x_{i}\right)$. Following Borchers et al. (2009), we assume unbiased distance estimation with gamma errors, that is, $E\left(y_{ik}|x_{i}\right)=d_{k}\left(x_{i}\right)$ and

(10) $$\begin{array}{ccc} & & \;f_{Y|X\Omega }\left(Y|X,\Omega ;\gamma \right)\\ & & \;\;=\prod_{i=1}^{n}\prod_{k=1}^{m_{i}}{\left\{{\left[\frac{d_{k}\left(x_{i}\right)}{\alpha }\right]}^{\alpha }\Gamma \left(\alpha \right)\right\}}^{-1}y_{ik}^{\alpha -1}\exp\left(-\frac{\alpha y_{ik}}{d_{k}\left(x_{i}\right)}\right), \end{array}$$

where *y_ik_* is the radial distance measurement from observer *j* to cue *i*.

For the case without measurement error, we define $f_{Y|X\Omega }\left(Y|X,\Omega ;\gamma \right)$ to be 1 if Y=X, and zero otherwise.

**Figure 7:**
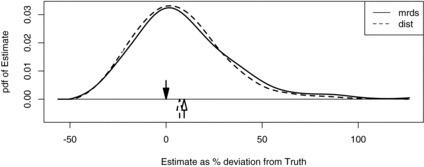
Smoothed simulated sampling distributions of estimated whale cue density when capture history and exact distances are observed (“mrds”) and when capture history and estimated distances are used (“dist”). The down arrow marks true density, the horizontal axis is percentage deviation from true density, and the up arrows are the means of the sampling distributions.

#### 4.3.2 Results

When distance measurement error is accommodated using an SECR model with estimated distance data, density is estimated to be 1.72 whale cues per hectare over the duration of the survey (CV = 18%), and detection probability at distance zero for the two detectors ($p_{k1}\left(0;\hat{\theta_{k}}\right)$, *k* = 1, 2) to be 1.0 (CV = 0.01%) and 0.30 (CV = 25%). When using an MRDS estimator in which distances are assumed to be error-free (as is the norm for such analyses), density is estimated to be 1.61 (CV = 17%) and $p_{k1}\left(0;\hat{\theta_{k}}\right)$, *k* = 1, 2 to be 1.0 (CV = 0.01%) and 0.30 (CV = 23%). Figure 6 shows the contours of estimated location of a whale detected only by detector 2, when observed distance is assumed error free and when it is estimated with measurement error.

Formulating the MRDS survey as an SECR estimation problem with distance measurement error provides a ready means of accommodating both measurement error and estimation of $p_{k1}\left(0;\theta_{k}\right)$—something that has to date not been done in analyses of DS data, with the exception of a model developed by Hiby and Lovell (1998) which used distance interval data rather than continuous distance measurements.

We conducted a simulation study (500 simulations) to investigate the effect of neglecting measurement error on density estimates, using the parameters estimated from the model that incorporates measurement error, with mean sample size of 70, and with error CVs of 12%, 32%, and 50%. Results for the 32% case are shown in Figure 7. On the 1987 NASS survey measurement error CV was estimated to be 32% compared to 12% on the 2001 survey—see Borchers et al. (2009). All estimators were found to be positively biased but those from the MRDS model were (in order of increasing measurement error CVs) larger by 14%, 34%, and 68%, respectively. Biases using the SECR model with measurement error were 7.7%, 7.0%, and 8.2%. As the model estimates six parameters from only 70 observations, we believe this to be small-sample bias.

## 5. DISCUSSION

We have shown that DS and CR are special cases of a more general class of spatial sampling model that uses detection locations to assist in estimating detection probability, and hence density. We have also shown that in the case of CR surveys, supplementing data on locations of captures with data on animal location (albeit noisy or incomplete) can substantially improve inference, particularly when designs are not optimal. Indeed, when MRDS surveys are considered as SECR surveys, most have the worst possible design (detectors at the same location) and inference about density from them would be impossible without the additional location data.

In the case of DS surveys, the new class of model provides a ready means for incorporating measurement error into inference, with or without the conventional DS assumption of certain detection at distance zero. The general model also provides a framework for incorporating into SECR surveys the point independence (Laake 1999; Innes et al. 2002; Borchers et al. 2006) and limiting independence (Buckland, Laake, and Borchers 2009) methods developed in the DS literature, as a means of reducing bias due to unmodeled heterogeneity.

### 5.1 Model Extensions

One topic that we have skirted, for lack of space, is how covariate data is incorporated into the models. Covariates can be incorporated in the density model D(x;φ) most naturally via a log link function, in the scale parameter of detection functions using a log link, and in the intercept parameter of detection functions using a logit link. Borchers and Efford (2008) and Royle et al. (2013b) contained SECR examples with a variety of explanatory variables and the former includes individual random effects. Marques and Buckland (2003) dealt with explanatory variables for DS models, and Borchers, Zucchini, and Fewster (1998) dealt with them for MRDS models.

We have also not covered any detail of how NHPP or other models that involve nonuniform animal distribution might be implemented. Although animal distribution is typically not homogeneous in space, it is usual to assume uniform spatial distribution in DS analyses (as a consequence of random placement of detectors), but DS estimators usually use this assumption only to estimate detection probability (estimating density conditional on detection probability using design-based methods). They have been found to be relatively robust to violation of the assumption at this level (see Buckland et al. 2001). Other methods may not be. Johnson, Laake, and Hoef (2010) implemented DS with an NHPP and Royle et al. (2013b) implemented a Bayesian version of SECR with an NHPP, with log-linear dependence on environmental covariates in both cases. We believe there is a need for more flexible models that are not necessarily monotonic in their dependence on explanatory variables, and expect that these will be developed in the near future. This could be achieved using penalized regression splines, in a similar way to that in which Gimenez et al. (2006) used them to model nonmonotonic dependence of survival probability in an open-population capture–recapture model.

Bayesian and frequentist versions of SECR have been developed in parallel by different authors. Bayesian inference tends to be particularly useful in the presence of latent variables or random effects—and animal locations are latent variables in SECR models. However, marginalization over locations involves a simple two-dimensional integral when locations are independent, making maximum likelihood inference straightforward. In this case, both approaches work well and it is largely a matter of personal preference which is used. Maximum likelihood estimation has to date proved to be much faster than the MCMC methods used for Bayesian inference, even when a random effect for unmodeled heterogeneity in detection probability is incorporated in the model. It seems likely that the Bayesian approach will come into its own when there is a more complicated latent variable structure—when there is dependence between latent variables, for example. In such cases, the marginalization required for maximum likelihood inference may become infeasible. We expect that models that do not involve independent distribution of animal locations (as NHPPs do) will soon be developed, as animals are often not independently distributed. A simple but common case is when animals occur in groups; in this case, animals within the group may not be detected independently of one another. This can often be dealt with by treating the group as the detection unit while simultaneously estimating mean group size if individual animal density is of interest, but in other cases models for spatial dependence may be required.

### 5.2 Robustness and Diagnostics

The robustness of estimators within the class of models developed in this article to failures of assumptions is likely to be case-specific. DS point estimators of density tend to be robust to failure of the assumption of independent uniform animal distribution (see Buckland et al. 2001, p. 36), although interval estimators are not. Efford, Borchers, and Byrom (2009a) found SECR point and interval estimators with multi catch traps to be robust to failure of assumptions of independence and uniformity (see Table 4, p. 266), and also found density estimates to be little affected by the form assumed for the detection function.

Goodness-of-fit diagnostics are well developed for DS detection function estimators, using observed locations (see Buckland et al. 2004, pp. 385–389). Similar diagnostics when locations are not observed remain to be developed (for both DS and SECR estimators). Borchers and Efford (2008) proposed a Monte Carlo goodness-of-fit test based on scaled deviance for the overall fit of SECR models but this does not distinguish between lack of fit of the animal density model and lack of fit of the detection model. This is an area that would benefit from further research.

### 5.3 Animal Movement

The methods of this article assume a single location (activity center) for each animal over the whole survey, but this does not imply or require that animals do not move during the survey. Nor does it require that movement between occasions on a multi occasion survey (*S* > 1) be modeled; providing that either (a) single- or multi-catch traps are used, or (b) occasions are long enough that the distribution of points that an animal visits over the duration of an occasion is the same as that over the duration of the whole survey. In the former case, there is no information on animal movement within occasions and the location is by definition the center of activity across occasions. In the latter case, the center of activity across occasions is identical to that within occasions. If proximity detectors are used and (b) above does not hold, then the detection functions within occasion will differ from those across occasions (typically having shorter ranges for shorter occasions). Models that do not allow for this are misspecified and may produce biased estimates. This problem can usually be avoided by having a design with occasions that are sufficiently long.

When activity centers move between occasions, an additional model layer for activity center movement will be required in general. The simplest such model is probably one in which the activity centers on each occasion are independent random effects with mean equal to an animal’s activity center across all occasions. But we believe that this will not be an adequate model in many applications, because activity centers on consecutive occasions are likely to be correlated. If activity centers are observed on some (but not all) occasions, the methods of Langrock and King (2013) and of references therein may be useful for modeling activity centers that were not observed, conditional on those that were. (If animal activity centers are the same for all occasions and some but not all are observed, the likelihood is like Equation (4), but with integration over only those centers that were not observed.)

### 5.4 Recapture Uncertainty

A final important issue that remains to be resolved for this class of model, and indeed for many CR models of any sort, is how to deal with uncertain recapture identification, as this can be fraught with uncertainty when animals are not physically tagged. This general problem was addressed by Link et al. (2010) for example, while Bonner (2013) and work referenced therein addressed the issue when there are multiple sources of individual identification. None of these methods explicitly use location information and we expect that methods that use location data to quantify the probability that detections are recaptures will be useful, as they were in the MRDS analysis of Hiby and Lovell (1998).

## APPENDIX A: VARIETIES OF *P* Ω ∣ *X*; θ

Multi-catch traps detain animals until the end of the sampling occasion in which they are trapped (and do not fill up). Proximity detectors are detectors that do not detain animals and therefore allow captures of the same animal on different traps within occasions. In some proximity detector applications, it is possible to detect the same animal more than once at the same detector. In this case, either binary capture histories of the sort used in the body of this article can be used or the capture frequency of each animal at each trap on each occasion can be recorded.

In the case of multi catch traps, all but one of ω_*i*1*s*_, …, ω_*iKs*_ are zero and Pr$\left(\omega_{is}|\omega_{i\cdot s}=1;\theta \right)$ is a multinomial distribution with index 1 and probabilities $p_{ks}\left(x_{i};\theta \right)/$ $\sum_{k}p_{ks}\left(x_{i};\theta \right)$ (*k* = 1, …, *K*). Modeling $p_{ks}\left(x_{i};\theta \right)$ using a competing hazard formulation (see Borchers and Efford 2008), $p_{ks}\left(x_{i};\theta \right)=r_{ks}\left(x_{i};\theta \right)p_{\cdot s}\left(x_{i};\theta \right)$, where $r_{ks}\left(x_{i};\theta \right)$ is defined as $h_{ks}\left(x_{i};\theta \right)/$ $h_{\cdot s}\left(x_{i};\theta \right)$, the relative hazard of detection at trap *k* on occasion *s* for an animal at $x_{i}$, $h_{ks}\left(x_{i}\right)$ is the detection hazard at trap *k* and $h_{\cdot s}\left(x_{i}\right)=\sum_{k}h_{ks}\left(x_{i}\right)$ is the total hazard on the occasion. Hence, $\sum_{k}p_{ks}\left(x_{i};\theta \right)=p_{\cdot s}\left(x_{i};\theta \right)$ and the multinomial probabilities are $r_{1s}\left(x_{i};\theta \right),...,r_{Ks}\left(x_{i};\theta \right)$.

In the case of proximity detectors with binary ω_*iks*_, Pr$\left(\omega_{is}|\omega_{i\cdot s}=1;\theta \right)$ is written as $\prod_{k=1}^{K}B\left(\omega_{iks},p_{ks}\left(x_{i};\theta \right)\right)/p_{\cdot s}\left(x_{i};\theta \right)$. In the case of proximity detectors with frequency data in which ω_*iks*_ is the frequency of detection on detector *k* on occasion *s*, Royle et al. (2009b) proposed a Poisson model for ω_*i* · *s*_, such that Pr$\left(\omega_{is}|\omega_{i\cdot s}=1;\theta \right)$ is $\prod_{k=1}^{K}\text{Po}\left(\omega_{iks},\lambda_{0}p_{ks}\left(x_{i};\theta \right)\right)/p_{\cdot s}\left(x_{i};\theta \right)$, where Po(*x*, λ) is a Poisson distribution with parameter λ.

With binary capture histories, Equation (2) reduces to Equation (A.1) below for proximity detectors when *K* = 1 and it reduces to Equation (A.2) with either kind of detector when *S* = 1.

(11) $$\begin{array}{ccc} P_{\left(K=1\right)}\left(\Omega |X;\varphi ,\theta \right) & = & \prod_{i=1}^{n}\frac{\prod_{s=1}^{S}B\left(\omega_{i1s},p_{1s}\left(x_{i};\theta \right)\right)}{p_{\cdot }\left(x_{i};\theta \right)}\\ P_{\left(S=1\right)}\left(\Omega |X;\varphi ,\theta \right) & = & \prod_{i=1}^{n}\frac{\prod_{k=1}^{K}B\left(\omega_{ik1},p_{k1}\left(x_{i};\theta \right)\right)}{p_{\cdot }\left(x_{i};\theta \right)}. \end{array}$$

These equations have the same form as the conditional likelihood of Huggins (1989). Equation (A.1) corresponds to the conventional CR case—in which there is usually more than one trap but all traps together are treated as a single composite trap, effectively with one location.

## SUPPLEMENTARY MATERIALS

Conventional point transect likelihood as a special case; Derivation of random effect TDOA distribution; Details of the R library admbsecr.
